# α-Synuclein Reactive Antibodies as Diagnostic Biomarkers in Blood Sera of Parkinson's Disease Patients

**DOI:** 10.1371/journal.pone.0018513

**Published:** 2011-04-25

**Authors:** Kiran Yanamandra, Marina A. Gruden, Vida Casaite, Rolandas Meskys, Lars Forsgren, Ludmilla A. Morozova-Roche

**Affiliations:** 1 Department of Medical Biochemistry and Biophysics, Umeå University, Umeå, Sweden; 2 P.K. Anokhin Institute of Normal Physiology, RAMS Moscow, Russia; 3 Departments of Molecular Microbiology and Biotechnology, Institute of Biochemistry, Vilnius University, Vilnius, Lithuania; 4 Department of Pharmacology and Clinical Neuroscience, Umeå University, Umeå, Sweden; National Institutes of Health, United States of America

## Abstract

**Background:**

Auto-antibodies with specificity to self-antigens have been implicated in a wide variety of neurological diseases, including Parkinson's (PD) and Alzheimer's diseases, being sensitive indicators of neurodegeneration and focus for disease prevention. Of particular interest are the studies focused on the auto-immune responses to amyloidogenic proteins associated with diseases and their applications in therapeutic treatments such as vaccination with amyloid antigens and antibodies in PD, Alzheimer's disease and potentially other neurodegeneration ailments.

**Methodology/Principal Findings:**

Generated auto-antibodies towards the major amyloidogenic protein involved in PD Lewy bodies – α-synuclein and its amyloid oligomers and fibrils were measured in the blood sera of early and late PD patients and controls by using ELISA, Western blot and Biacore surface plasmon resonance. We found significantly higher antibody levels towards monomeric α-synuclein in the blood sera of PD patients compared to controls, though the responses decreased with PD progression (*P*<0.0001). This indicates potential protective role of autoimmunity in maintaining the body homeostasis and clearing protein species whose disbalance may lead to amyloid assembly. There were no noticeable immune responses towards amyloid oligomers, but substantially increased levels of IgGs towards α-synuclein amyloid fibrils both in PD patients and controls, which subsided with the disease progression (*P*<0.0001). Pooled IgGs from PD patients and controls interacted also with the amyloid fibrils of Aβ (1–40) and hen lysozyme, however the latter were recognized with lower affinity. This suggests that IgGs bind to the generic amyloid conformational epitope, displaying higher specificity towards human amyloid species associated with neurodegeneration.

**Conclusions/Significance:**

Our findings may suggest the protective role of autoimmunity in PD and therefore immune reactions towards PD major amyloid protein – α-synuclein can be of value in the development of treatment and diagnostic strategies, especially during the early disease stages.

## Introduction

Protein aggregation leading to amyloid deposition in the brain is implicated in the pathology of a number of neurodegenerative diseases such as Parkinson's (PD), Alzheimer's (AD), prion diseases and others [1], [2], [3], [4]. The amyloid deposits can be built up from sequentially and structurally diverse proteins and peptides, however when they self-assembled into amyloid fibrils, the latter display a common denominator - a cross-β-sheet core stabilized by a dense inter- and intra-molecular hydrogen bond network between the amino and carbonyl groups of polypeptide chain. Remarkably, the amyloid species display also a generic conformational epitope, reflecting their common architecture, which is recognized by the oligomer- and fibril-reactive immunoglobulin antibodies, though the molecular basis of this recognition is still debated [5], [6] The amyloid reactive antibodies, due to their unique specificity, have significant diagnostic and therapeutic potential for patients with amyloid-associated diseases. It has been suggested that autoimmune reactions towards specific proteins and their self-assembled complexes involved in disease pathology can be used as sensitive biomarkers of neurodegeneration in both AD and PD [7], [8], [9]. Immunoglobulin G (IgG) antibodies that recognize a conformational epitope(s) of the fibrils of Aβ peptide and other polypeptides, but do not recognize these components in their native non-fibrillar states, have been found even in healthy human sera [10]. This suggests that autoimmune reactivity can play an important role as potential amyloid clearance mechanism in both health and disease. Recently, the vaccine development has received great attention among therapeutic approaches in AD treatment [11], [12], [13], [14]. This includes both passive vaccination with antibodies [11], [12] and active vaccination with Aβ_42_ peptide [14] and its pre-aggregated forms [13]. The discovery by Schenk *et al.* [14], that mice immunized with Aβ_42_ peptide has significantly reduced amyloid deposits and neuritic pathology, was the most intriguing finding with regard to AD treatment. This approach showed that it is possible not only to slow the progression of amyloid deposits, but even reverse them. The active immunization, which generates antibodies neutralising amyloid toxicity [13], [15], has opened an avenue to treat AD in humans. However, the active anti-Aβ vaccination trial in patients with mild-to-moderate AD was prematurely halted when 6% of inoculated individuals developed aseptic meningoencephalitis [16]. In our research we examined the autoimmune reactions towards the key component of PD amyloid deposits – α-synuclein and its aggregated species and discussed their role in neurodegenerative processes.

PD is the second most common neurodegenerative disorder with a prevalence progressively increasing from 0.6 to 3.5% during aging from 65 to 89 y.o. as shown in collaborative surveys conducted in France, Italy, Spain and Netherlands [17] as well as the study performed in the Northern Sweden demonstrated a cumulative incidence (lifetime risk) being close to 3% among the population with up to 89 years of age [18]. PD is characterized by resting tremor, bradykinesia and muscular rigidity. Most of PD cases (90–95%) are sporadic, whereas familial PD constitutes only 5–10%. Dopamine neurons in the *substantia nigra* region of PD brain are characterized by intracellular amyloid deposits of α-synuclein known as Lewy bodies [19]. PD progression is commonly associated with death of dopaminergic neurons and up to 70–80% of neurons in the *substantia nigra* may be already dead by the time when the clinical symptoms become obvious [20].

α-synuclein is relatively abundant in the brain under non-pathological conditions. It is a natively unfolded protein present mostly in the cytosol. It plays an essential role in synaptic transmission and synaptic plasticity by augmenting transmitter release from the presynaptic terminal [21]. There is substantial evidence that the conversion of α-synuclein from its soluble into the aggregated insoluble form is one of key events in the pathogenesis of PD [22], [23]. *In vitro* α-synuclein can also self-assemble into ordered amyloid species, which are similar to those found in Lewy bodies or formed by other amyloid polypeptides, such as Aβ, thus confirming that α-synuclein is naturally amyloidogenic protein [24]. Lewy bodies grow intracellularly, but upon the neuronal death or damage of axons in the *substantia nigra*, the aggregated species of α-synuclein are released into the extracellular space [19], [23]. Among them, soluble oligomers are primarily toxic to the cells [25], [26], [27]. It is important to note, that recently it has been shown in *Saccharomyces cerevisiae* [28], *Caenorhabditis elegans* [29] and *Drosophila melanogaster* [30] models that α-synuclein, if over expressed, itself can be also damaging to cells, causing oxidative stress, vesicle trafficking defects and impairment of chaperon and ubiquitin-proteasome systems. Both monomeric and oligomeric α-synuclein have been found in the cerebrospinal fluid (CSF) and serum of PD patients [31], [32], [33], [34], [35], [36], as apparently α-synuclein and even its aggregated species can cross the blood brain barrier. In this study we assessed if the immune reactivity towards α-synuclein monomeric and amyloid forms in the blood sera can be detected and possibly serve as a biomarker in PD diagnostics and prognostics; we set our primary focus on the initial PD stages when the clinical diagnostics is particularly challenging.

## Results

### α-synuclein amyloid formation

**Figure 1:**
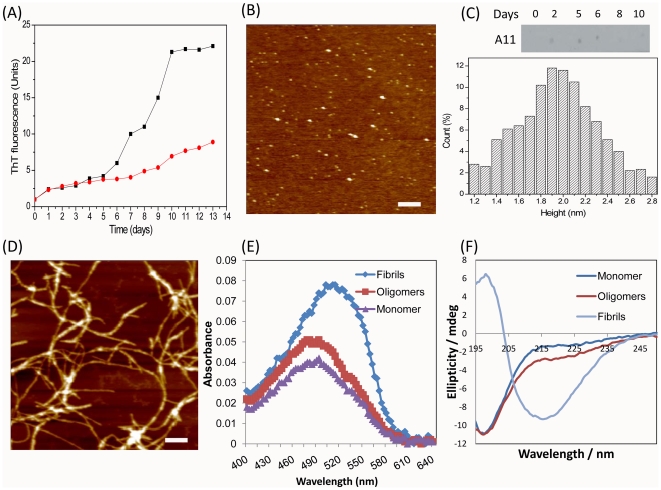
Amyloid properties of α-synuclein. (A) Kinetics of amyloid formation monitored by thoflavin T fluorescence at α-synuclein concentrations of 0.71 mM (black squares and line) and 0.21 mM (red circles and line). (B) AFM images of α-synuclein oligomers. (C) Top: dot blot analysis of interactions of anti-oligomeric A11 antibodies with the amyloid containing aliquots; days of amyloid incubation are indicated. Bottom: the distributions of the *z*-heights of α-synuclein oligomers measured by AFM cross-section analysis. (D) AFM images of α-synuclein fibrils. Scale bars in *x,y*-plain equal to 500 nm (B,D). (E) Congo red binding to α-synuclein monomeric, oligomeric and fibrillar species as denoted by corresponding color coding. (F) CD spectra of monomeric, oligomeric and fibrillar α-synuclein as denoted by corresponding color coding.

In order to produce amyloid species, α-synuclein was incubated at 37°C with continuous agitation (see Materials and Methods). Aliquots were collected at regular intervals and subjected to thioflavin T binding assay, assessing the formation of typical amyloid cross-β-sheet structure, which is manifested in an increase of fluorescence intensity measured at 485 nm (Figure 1A). The kinetics of amyloid formation at 0.71 mM concentration of α-synuclein displayed a sigmoidal shape and the samples containing mature amyloid fibrils were collected on 10^th^ day of incubation, corresponding to the plateau region (Figure 1A). The samples containing oligomers were collected at the end of the lag-phase of ca. 6 days, during which we observed small but steady fluorescence increase upon incubation of the samples with both 0.71 and 0.21 mM protein concentrations. In order to exclude the presence of some spontaneously formed fibrils in the oligomeric fractions, the oligomers produced at lower 0.21 mM concentration of protein were taken for further experiments.

The morphology of aggregated species was examined by AFM imaging as shown in Figures 1B, 1D. The oligomers significantly populated in the sample after 6 days of incubation displayed a round-shaped morphology with the wide distribution of heights from 1.2 to 2.8 nm and centered at ca. 2 nm as measured in the AFM cross-sections (Figure 1C); the fibrils have not been observed in this sample (Figure 1B). After 10 days of incubation we observed an extensive network of mature amyloid fibrils (Figure 1D), characterized by significant length of up to 2–5 µm and height in AFM cross-sections of 8–10 nm, indicative of inter-winding of a few amyloid protofilaments.

The interaction of amyloid species with generic A11 antibodies reactive towards the amyloid oligomers were examined by the dot blot analysis and shown in the top panel in Figure 1C. Indeed, we observed that A11 antibodies interact with the oligomeric species populated during days 2 to 6 in the 0.21 mM sample, but the binding subsides in the aliquots, containing mature fibrils collected on day 8 and 10 from the 0.71 mM sample, even though the latter has a higher quantity of amyloids.

The oligomers and fibrils of α-synuclein bind also amyloid specific dye - Congo red, which is reflected in a long-wavelength shift and increase of the dye absorbance spectra compared to the control measurement of Congo red spectrum in the presence of monomeric α-synuclein (Figure 1E). The Congo red binding to oligomers caused only ca. 2 nm red shift (the long-wavelength shift is more obvious in the differential spectrum, i.e. when the spectrum of Congo red alone is subtracted from the spectrum of Congo red in the presence of the target oligomers [37], data not shown) and small increase of the spectrum, while upon interaction with fibrils the effect was much more pronounced, leading to the ca. 2 fold increase of absorbance and ca. 30 nm red spectral shift (Figure 1E). The development of β-sheet core in the amyloid species were monitored also by the far UV circular dichroism (CD) spectra (Figure 1F). The monomeric protein possesses a spectrum typical of a disordered polypeptide chain, characterized by the presence of the intensive minimum in the 197 nm region. Small broad shoulder between ca. 215 and 235 nm emerges in the oligomer-containing sample indicating a slightly more ordered secondary structure than the natively unfolded monomeric α-synuclein and very low if any β-sheet content. With this respect, the spectrum of oligomeric sample resembles those of oligomers produced at 20 to 100 mM NaCl concentrations and pH 7.5, 37°C, 600 ppm shaking in [38]. By contrast the fibrillar sample of α-synuclein displays a very characteristic β-sheet spectrum with a broad band centered at ca. 216 nm.

As cytotoxicity is viewed as the most harmful manifestation of amyloid assemblies, we examined the effect of α-synuclein amyloid species on the viability of SH-SY5Y cells (Figure S1). The oligomers characterized above (Figure 1B) reduced cell viability by ca. 30% to 50% after 24 and 48 hours of co-incubation with the cells, respectively, while the fibrillar species (Figure 1D) did not induce noticeable cytotoxicity.

### Autoimmune response towards monomeric α-synuclein

The presence of auto-antibodies against α-synuclein monomeric species in the peripheral blood sera of each individual from the PD patient and control groups was assessed by ELISA and immunoblot detection methods. Prior subjecting to the immunological screening the freshly dissolved α-synuclein was examined by SDS-PAGE, which shows a single band corresponding to monomeric protein (Figure S2). By conducting AFM imagining we also did not observe any larger aggregated species in the samples with monomeric α-synuclein (data not shown).

**Figure 2:**
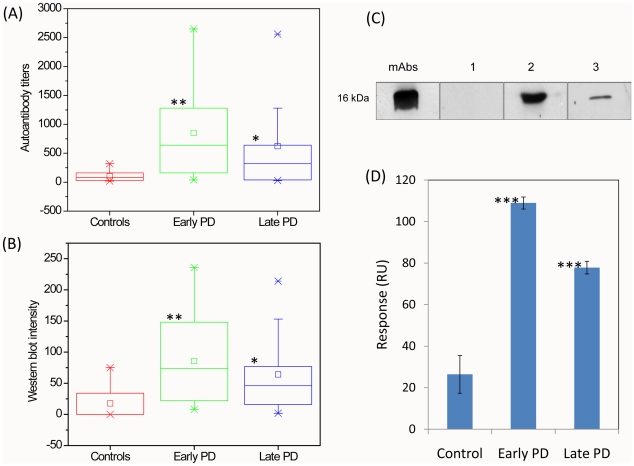
Immune responses towards α-synuclein monomer in the blood sera of controls, early and late PD patients. Box-plots showing statistical distributions of immune responses to α-synuclein measured by ELISA (A) and Western blotting (B). The antibody titers (A) and Western blot band densities (B) are shown along *y*-axis and the groups subjected to analysis - along *x*-axis. Boxes include from 25% to 75% of all immune responses; central squares indicate the mean and line drawn crossed the box – the median values for each group; whiskers indicate the distribution from 5% to 95%, while small crosses correspond to remaining 10%. (C) Representative Western blots showing interactions with monomeric α-synuclein of monoclonal antibodies (mAbs) and sera IgGs from selected control (1), early PD (2) and late PD (3) individuals. (D) Biacore analysis of the interactions with α-synuclein of pooled IgGs from controls, early and late PD patients. Surface plasmon resonance responses in relative units are shown in *y*-axis. ***P<0.0001, **P<0.007 and *P<0.05.

**Figure 3:**
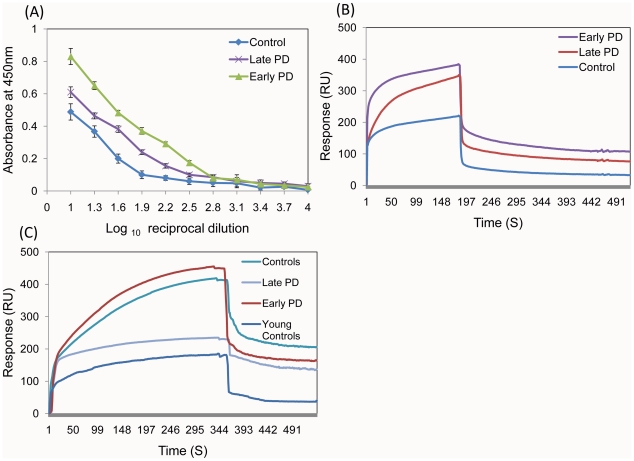
Interaction of α-synuclein species with the sera antibodies of PD patients and controls. ELISA titration curves corresponding to the interactions of monomeric α-synuclein with IgGs from the blood sera of representative control, early PD and late PD individuals as denoted in the caption (A). Biacore sensorgrams reflecting the biding of α-synuclein monomer (B) and fibrils (C) with IgGs from the pooled sera of control, young control (C), early PD and late PD individuals as denoted in the captions.

The results of ELISA summarized in the box-plots are presented in Figure 2A; the representative ELISA titration curves are shown in Figure 3(A). These observations demonstrate that in healthy individuals the immune responses towards α-synuclein were at the cut-off level of ELISA, displaying a very narrow distribution of titers. By contrast, in early PD patients there was a significant increase of IgG reactivity towards α-synuclein (*P*<0.0001), accounting for rise by ca. 8 fold of mean and median values of titers compared to these values in controls. There was a wider distribution of responses in this group than in controls with ca. 70% of patients displaying high immune reactivity towards α-synuclein. It is important to note that 3 individuals displayed particularly high responses, with up to ca. 25 fold enhancement in the IgG - α-synuclein reactivity as estimated by their titers. In the blood sera of late PD patients there was also an increase of immune-reactivity towards α-synuclein (*P*<0.007) compared to controls, with ca. 6 fold higher mean and ca. 4 fold higher median, respectively, and with ca. 58% patients displaying high immune reactivity, but the values of their titers were lower than in the early PD group.

The analysis of immune-reactivity in the blood sera of patients and controls by immunoblotting is summarized in the box-plots in Figure 2B. The samples of freshly dissolved α-synuclein were subjected to Western blot analysis and subsequently treated with the blood sera. The representative Western blots are shown in Figure 2C, indicating the strong interaction of α-synuclein with monoclonal antibodies used as a reference; the absence of recognition in the blood sera of representative individual from the control group, strong recognition of α-synuclein by IgGs in the PD patient serum from the early PD group and weaker binding in the specimen from the late PD patient. The box-plots demonstrate the same tendency as the results obtained by using ELISA: early PD patients were characterized by a significant increase in IgG reactivity towards α-synuclein compared to controls (*P*<0.0001) with ca. 5 and ca. 10 fold increase of mean and median values, respectively, as estimated by the density of Western blot bands, and with 63% of individuals exhibiting the high responses. In the late PD group the immune responses subsided, showing ca. 4 and 6 fold enhancement in mean and median, respectively (*P*<0.007), compared to controls and with ca. 58% of patients showing high level of antibodies. As Western blot method can be more sensitive than ELISA, enabling to register the presence of up to picomolar concentration of antibodies, we have observed some weak responses towards α-synuclein in 4 individuals in the control group, which is reflected in a wider distribution of readings in the corresponding box-plot compared to the ELISA data (Figure 2B). There were no significant correlations between the immune responses in PD patients and controls with their age or gender as assessed by both ELISA and Western blot analyses.

**Figure 4:**
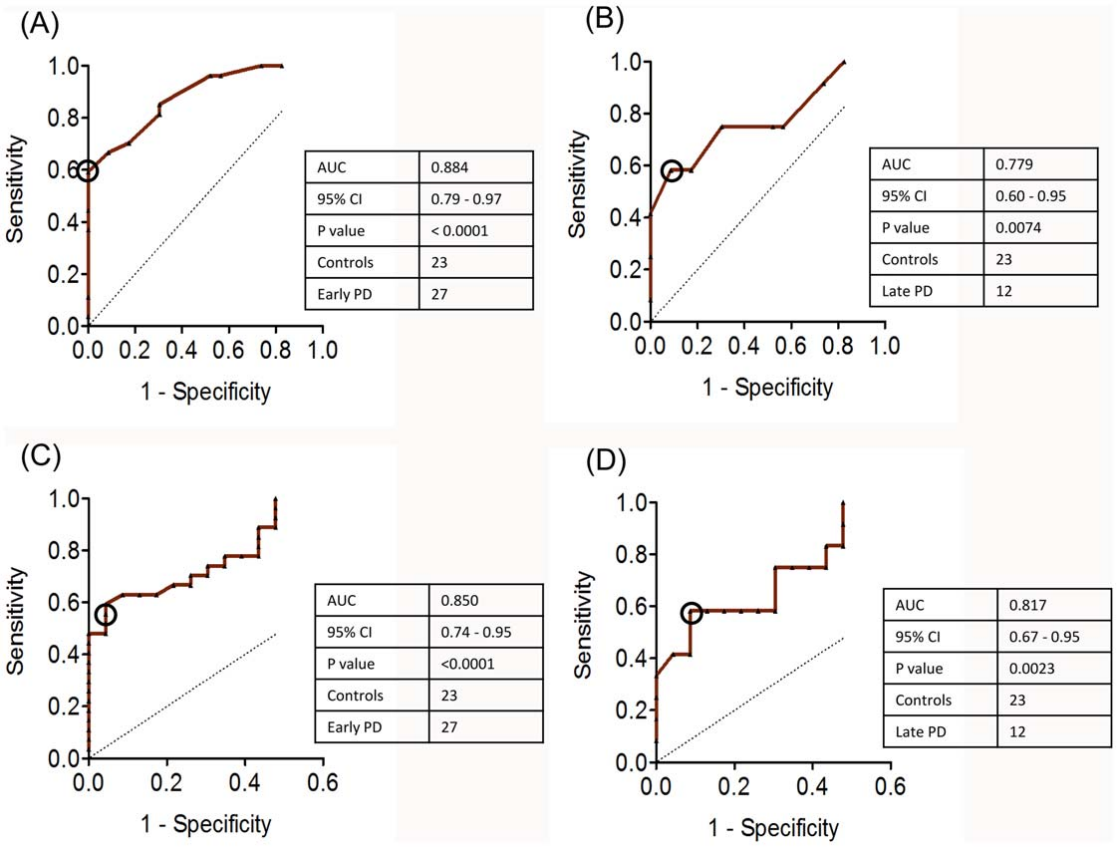
Assessment of diagnostic value of autoimmune responses to α-synuclein in PD patients. Receiver operating characteristic (ROC) curves comparing the autoimmune responses towards α-synuclein between early PD (true positives) and controls (true negatives) determined by ELISA (A); between late PD and controls determined by ELISA (B); between early PD and controls determined by Western blot (C) and between late PD and controls determined by Western blot (D). The dotted line indicates the results when the parameter in question has no diagnostic value (AUC = 0.5). The optimum cutoff values (Youden index) are shown by the open circles. The tables summarize AUC, 95% confidence interval (CI), P value, number of controls and patients.

The diagnostic potentials of the ELISA and Western blot measurements in discriminating the higher autoimmune responses in early / late PD patients compared to controls were also assessed statistically by the receiver operating characteristic (ROC) analysis as shown in Figure 4. The areas under the ROC curves (AUC) for the autoimmune reactivity determined by ELISA in early and late PD patients compared to controls were 0.884 (95% confidence interval of 0.79 to 0.97) and 0.779 (0.6–0.95), respectively. The AUC values calculated from the corresponding ROC curves for the Western blot data were 0.85 (0.74–0.95) for early PD vs controls and 0.817 (0.67–0.95) for late PD vs controls, respectively. This indicates that the autoimmune responses to α-synuclein have a high diagnostic value both in early and late PD patients. For the autoimmune responses determined by ELISA in early PD cases vs controls, the optimal cut-off value was set at >480 (J = 0.59). At this division, 59% of the early PD cases were true positives with 0% false positives. For the Western blot data the corresponding cut-off value of >52 (J = 0.55) identified 59% as true positives in early PD group with 4% (1 of 23 controls) false positives. For the immune reactivity in late PD patients vs. controls assessed by ELISA and Western blot, the optimal cut-off values were >280 (J = 0.49) and >45 (J = 0.49), respectively, indentifying 58% true positives and 8.7% (2 of 23 cases) false positives in both experimental set ups. The AUC for the immune reactivity towards α-synuclein in early vs late PD patients evaluated by both ELISA and Western blot analysis were not significantly greater than 0.5 and the ROC curves were close to diagonals, respectively (data not shown).

To provide an additional support to our findings we have subjected the pooled purified IgGs from blood sera of controls, early and late PD patients to Biacore surface plasmon resonance analysis, by using the Biacore chips coated with α-synuclein monomers (Figure 2D); the Biacore sensograms are presented in Figure 3(B). Polyclonal antibody responses are given in relative units measured after washing, when the signal reached an equilibrium value, to ensure that only strong binding of α-synuclein-specific antibodies is registered vs. non-specific interactions with the Biacore chip by other pooled sera IgGs. Consistently with the ELISA and immunoblot data, the level of IgG binding to α-synuclein was significantly higher both in the blood sera of early (*P*<0.0001) and late (*P*<0.05) PD patients compared to controls, however, the late PD group showed decreased level of antibodies compared to early PD patients.

### Autoimmune responses towards α-synuclein amyloid oligomers and fibrils

The blood sera from PD patients and healthy controls were also examined on the presence of immune responses towards α-synuclein oligomers and fibrils. We have not observed any specific responses towards amyloid oligomers characterized above, neither in majority of early and later PD patients nor in controls, by using ELISA (data not shown). There were 2 exceptions in the control group characterized by the significant titers of IgGs towards oligomers of ca. 2500 and 650; among 27 early PD patients only 1 individual showed response with ca. 2500 and 2 - with ca. 650 titers, respectively, and in the late PD group 2 patients out of 12 exhibited titers of ca. 650. Examination of the pooled sera from early and late PD patients and controls by Western blot analysis did not show substantial immune reactivity (data not shown), confirming the ELISA results on the lack of immune responses towards oligomers in the vast majority of healthy and PD individuals. In control experiments we examined the interactions of the pooled sera IgGs of PD patients and healthy individuals with the oligomers of α-synuclein (0.21 mM sample) produced during six days of incubation at five pH values in the range from 6 to 8 with 0.5 pH increment in each sample. As pH can affect the conformational properties of oligomers, this enabled us to assess a wider variety of these potentially heterogeneous species. We did not observe significant interactions between the oligomeric samples and pooled IgGs in either of these cases (data not shown).

**Figure 5:**
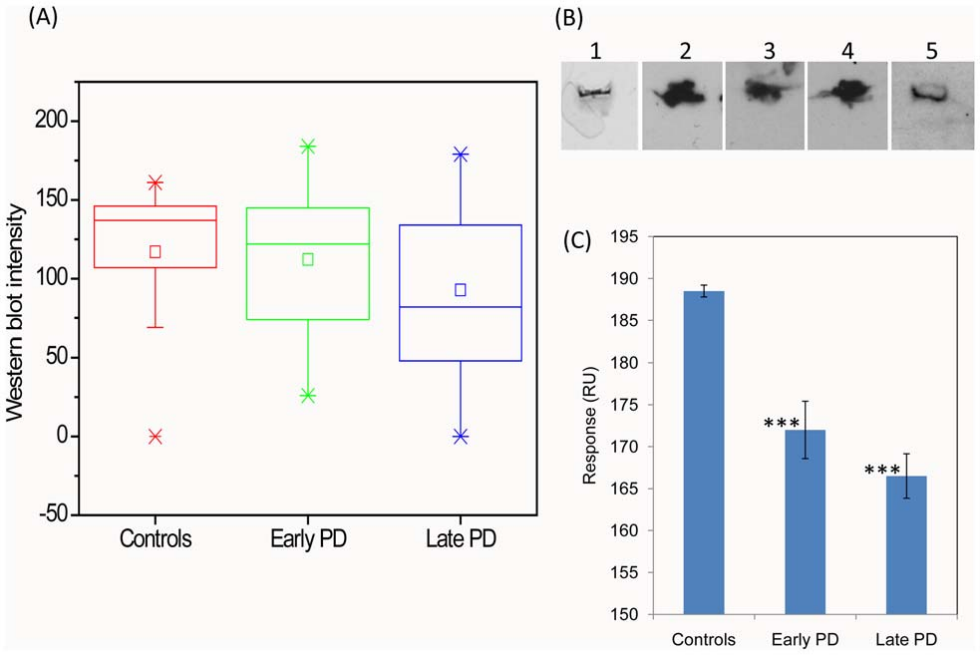
Immune responses towards amyloid fibrils of α-synuclein in the blood sera of controls, early and late PD patients. (A) Box-plots showing statistical distributions of immune responses measured by Western blotting. The descriptions of box-plots are the same as in Figure 2B. (B) Representative native gel band corresponding to amyloid fibrils (1) and Western blots bands showing interactions of α-synuclein fibrils with anti-fibrillar antibodies (2), with sera IgGs from selected control (3) as well as early PD (4) and late PD (5) individuals. (C) Biacore analysis of the interactions with amyloid fibrils of pooled IgGs from the blood sera of controls, early and late PD patients. Surface plasmon resonance responses in relative units are shown in *y*-axis. ***P<0.0001.

In order to measure the immune reactivity of sera IgGs towards amyloid fibrils we used Western blot analysis (Figure 5A,B). The amyloids were loaded on the native gel; the higher molecular weight species remained on the top of the gel (representative band is shown in Figure 5B, left) and, thus, were separated from the remaining low molecular weight and monomeric α-synuclein species entering the gel. Consequently, Western blot analysis was carried out with these species. The Western blots presented in Figure 5B demonstrate the clear recognition of amyloid fibrils by specific anti-fibrillar IgGs [6] selected as a reference (band 2) and by the sera IgGs of representative individuals from the control, early and late PD groups (bands 3–5), respectively.

The summary of Western blot analysis is presented in the box-plots in Figure 5A for each examined group, demonstrating substantial immune responses towards α-synuclein amyloid fibrils in all of them. There were no statistically significant differences in the distributions of immune reactivities between the groups with the mean and median values equal to 117/137, 112/122 and 93/97 for control, early PD and late PD groups, respectively. Both PD groups showed the wider distributions of responses than the control group. In the control group 4 out of 23 individuals were characterized by rather low anti-fibrillar IgG reactivity. The ROC analysis of the immune responses to fibrils in early / late PD patients vs controls gave the AUC values not greater than 0.5 and ROC curves close to diagonals (data not shown). There were no significant correlations between the immune responses towards amyloid fibrils in PD patients and controls with their age or gender.

The amyloid fibrils of α-synuclein were used also as antigens in the Biacore experiments, in which they were placed on the Biacore chip and their interactions with the IgGs purified from the pooled blood sera of each studied group were monitored as described in Material and Methods. There was significant immune reactivity towards fibrillar antigens in all samples with a slight decrease by ca. 10% between the control and early / late PD groups, respectively (Figure 5C) (*P*<0.0001); the Biacore sensograms are presented in Figure 3(C). This is consistent with the trend of decreasing immune reactivity towards amyloid fibrils with progression of PD detected by the Western blot analysis.

### Cross-reactivity of anti-fibrillar IgGs with amyloid fibrils of different protein origin

It has been shown recently that fibril specific, conformation-dependent antibodies are able to recognize a generic epitope common to various amyloid fibrils [6] as well as the fibril-reactive purified antibodies immunostained amyloid deposits in human tissues and displayed *in vivo* reactivity in a murine model [10]. Therefore we examined here if anti-fibirllar IgGs found in PD patients and controls possess a similar cross-reactivity towards different fibrillar species and belong to this class of generic fibril specific antibodies.

**Figure 6:**
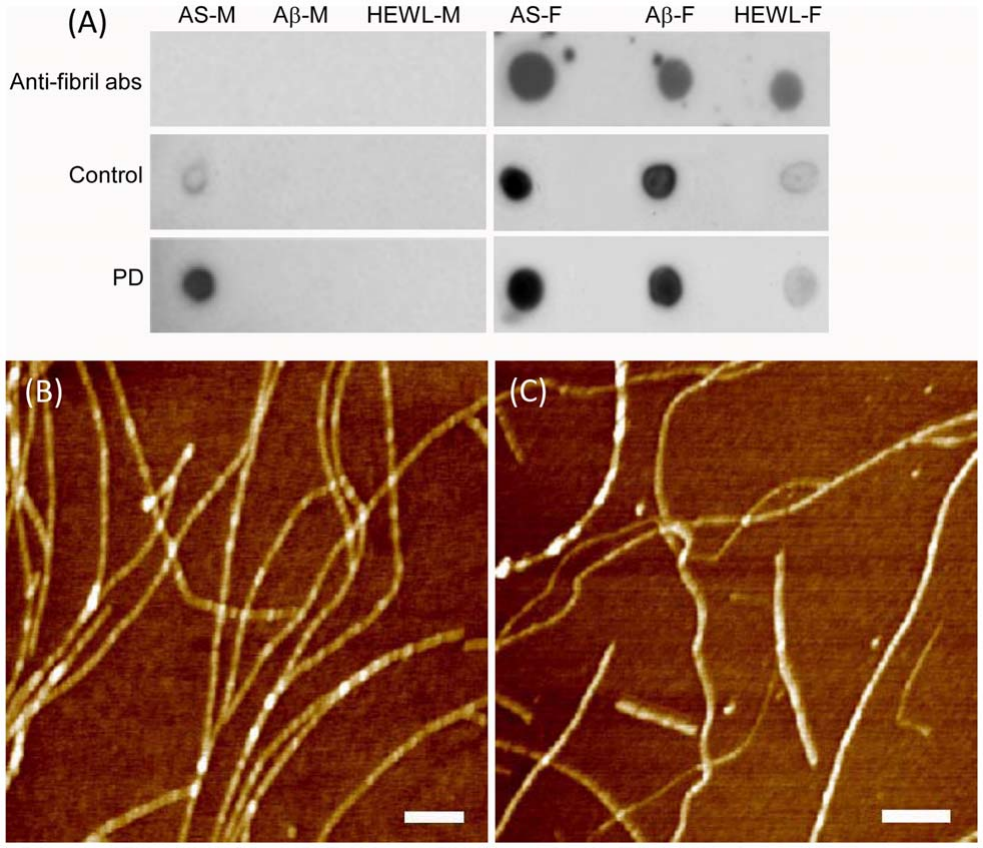
Immune reactivity of pooled IgGs from the blood sera of controls and PD patients towards monomers and amyloid fibrils of different polypeptides measured by dot blot analysis. (A) Dot blots demonstrating the immune reactivity towards monomers of α-synuclein (AS-M), Aβ peptide (Aβ-M) and hen egg white lysozyme (HEWL-M) are shown in the left panel and towards corresponding fibrils (AS-F, Aβ-F and HEWL-F) – in the right. The rows of dot blots from top to bottom show the interactions of antigens with added anti-fibrillar antibodies [6] and pooled IgGs from the sera of controls and all PD patients, respectively. AFM images of amyloid fibrils of Aβ peptide (B) and hen egg white lysozyme (C) subjected to dot blot analysis. Scale bars in *x,y*-plain equal to 500 nm.

Purified IgGs from the pooled blood sera of PD patients and controls were compared with regards to their reactivity towards fibrillar antigens of different protein origin, i.e. formed from α-synuclein, Aβ (1–40) peptide and hen egg white lysozyme (Figure 6A), by using dot blot analysis. Both Aβ (1–40) peptide and hen egg white lysozyme developed mature amyloid fibrils as described in Material and Methods and examined by AFM imaging (Figure 6B and 6C). Specific anti-fibrillar IgGs [6], used as a reference, recognized all types of selected fibrils (Figure 6A, right), but not corresponding monomers (Figure 6A, left). By contrast the pooled IgGs of all PD patients and controls, respectively, interacted with the fibrillar antigens of all selected polypeptides, though their reactivity with α-synuclein and Aβ peptide fibrils was much more pronounced than with the fibrils of hen egg white lysozyme. Consistently with the above reported results, the pooled IgGs of all PD patients and to a lesser extent IgGs of controls recognized monomeric α-synuclein, but not the monomers of Aβ peptide or hen egg white lysozyme (Figure 6A, left). This indicates that in blood sera of both PD patients and controls there are polyclonal IgGs reactive with conformational epitope of amyloid fibrils, targeting, however, primarily the fibrils associated with human neurodegenerative ailments (α-synuclein and Aβ peptide).

## Discussion

Auto-antibodies with specificity to self-antigens have been implicated in a wide variety of neurological diseases [7], [8], [39] including PD [9], [40], as they reflect the pathological changes occurring in the brain during neurodegeneration. By using a range of detection methods such as ELISA, Western blot and surface plasmon resonance by Biacore, we have shown that there is a substantial increase of immune responses to monomeric α-synuclein in early PD patients compared to controls, with 70% of individuals displaying high level of IgGs as estimated by ELISA and 63% - by Western blot analysis, respectively (Figure 2). The immune reactivity to α-synuclein decreased in late PD, which reflected in a lower number of patients exhibiting high immune responses, i.e. 58% as determined by both ELISA and Western blot, and also in the lower values of the responses. In both ELISA and Western blot screening we observed the robust ROC curves and AUC≥ca. 0.8, indicating that the autoimmune reactivity towards α-synuclein has a significant diagnostic value. Although the choice of cut-off values is dependent upon the importance of missing true positives vs. misdiagnosing false positives, the Youden index values calculated from the ROC curves would suggest that >480 and >52 cut-offs identify 59% true positives with a 0 and 4% (1 of 23 cases) false positive rate in early PD by using ELISA and Western blot, respectively, and the cut-offs of >280 and >45 for ELISA and Western blot analysis, respectively, identify 58% true positives vs. 8.7% (2 of 23 cases) false positives in late PD by both experimental approaches. No correlation with age or gender was found.

In previous study by Papachroni *et al.* [40] the enhanced level of multi-epitopic antibodies against α-synuclein was also detected in the blood sera of 65% of PD patients compared to controls by using Western blot analysis. However, the authors observed correlation primarily with familial PD, i.e. 90% of patients were positive for antibodies towards α-synuclein in familial PD vs 48% in sporadic cases. It is important to note, that these patients were characterized by ca. 2.5 of Hoehn and Yahr score, which corresponds to our late PD group, and consistent with lesser activity of their humoral immunity towards α-synuclein compared to early PD in our studies.

There is evidence that PD pathology engages humoral immunity, which is reflected in a detectable level of IgGs in PD brains, binding to dopaminergic neurons in a concentrated distribution at neuronal surfaces or co-localized with α-synuclein on Lewy bodies [41]. Moreover, initiation of humoral immunity in early onset PD may also be concomitant with inflammation [42], which plays a role in neurodegenerative diseases [43]. The major participant of PD - α-synuclein is an abundant neuronal protein expressed in many neurons, including dopaminergic neurons in the *substantia nigra*. Its fibrillar form is a main constituent of the Lewy bodies. The death of neurons or damage of axons and synaptic terminals could result in the release of soluble and aggregated forms of α-synuclein into the extracellular space. There is also evidence of physiological secretion of α-synuclein into extracellular environment [44]. Altered α-synuclein metabolism in the central nervous system can be manifested in the body fluids and as a result α-synuclein has been found in the CSF and plasma of PD patients [31], [33], [35], [36], [45]. It is interesting to note, that the decreased level of α-synuclein in CSF was found in the large scale screening of PD patients compared to AD patients and controls by Hong *et al.* [45] as well as an inverse correlation of α-synuclein content with Hoehn and Yahr score was observed by Tokuda *et al.* [32]. However, the results on α-synuclein content in plasma/sera are highly variable - Li *et al.* [36] presented a decreased plasma level of α-synuclein in PD compared to controls as measured by Western blotting, while Lee *et al.* [33] reported an increased plasma α-synuclein concentration in PD compared to controls by using ELISA. The variations can be related to the fact that cross-reactive species may involve both monomeric α-synuclein and its aggregated forms in these measurements.

In PD pathology the autoimmune reactivity can serve as a protective mechanism aimed to maintain the body homeostasis and eliminate intrinsic factors leading to its disbalance. As enhanced concentration of α-synuclein can be a significant aggregation prone factor, promoting its self-assembly into toxic amyloid oligomeric species and amyloid fibrils, the autoimmunity can have a potential protective power, reducing its concentration in the body fluids. Indeed, vaccination of transgenic mice with human α-synuclein led to production of highly reactive antibodies, which decreased accumulation of aggregated α-synuclein in neuronal cell bodies and synapses and consequently reduced neurodegeneration [46]. Furthermore, antibodies produced by immunized mice recognized abnormal α-synuclein associated with membrane and promoted the degradation of its aggregates. Similar effects were observed with an exogenously applied α-synuclein antibodies, indicating that they can be a powerful tool in reducing neuronal accumulation of α-synuclein aggregates [46]. In our studies the increased level of α-synuclein reactive IgGs found in early PD can fulfill the protective function reducing the concentration of α-synuclein in the blood sera. In the late PD group the level of anti-α-synuclein antibodies and their clearance power subsided, as disbalance in the body homeostasis can be overwhelming.

We have not found statistically significant immune responses towards oligomeric species in all studied groups, including controls, early and late PD patients. By contrast, we have observed substantial immune responses towards mature amyloid fibrils in the control group and subsiding, but still pronounced, reactivity in early PD and to a lesser extend in late PD as measured by Western blotting (Figure 5A). The consistent decrease of immune reactivity towards fibrils was detected by Biacore analysis in the pooled fractions of IgGs of the control, early and late PD groups, respectively (Figure 5C). The overall decreased reactivity of naturally occurring antibodies towards monomeric and fibrillar α-synuclein in late PD patients may be related to T-cell tolerance, when these cells become deficient in producing antibodies and providing help in clearance of these molecular species [47].

It is important to note, that amyloid-reactive IgGs are naturally present in the blood sera of healthy individuals, recognizing common conformational epitope of amyloid fibrils regardless of their protein composition [10], [48]. Consistently with this, we have found the cross-reactivity of IgGs pooled from the blood sera of PD patients and controls towards the amyloid fibrils of different protein origin (Figure 6A). Indeed, the pooled IgGs from both groups recognized the amyloid fibrils of α-synuclein, Aβ (1–40) peptide and even to a smaller extent hen egg white lysozyme fibrils as demonstrated by dot blot analysis. All types of fibrils exhibited rather similar morphology as shown by AFM imaging (Figure 1D, 6B, 6C), nonetheless they may have some sequence or species specificity, which led to decreased binding of IgGs to hen egg white lysozyme fibrils.

The fact that we did not observe statistically significant immune reactivity towards the oligomeric species of α-synuclein both in PD patients and controls, which are the most pathogenic compounds causing neuronal cell death (Figure S1), maybe related to the fact that they present transiently and rapidly inter-convert into larger pre-fibrillar or fibrillar species. Soluble oligomers are often transient, since they are consumed as fibrillization proceeds [25], [38], [49], [50]. *In situ* AFM analysis demonstrated that the assembly of globular oligomers precedes the formation of amyloid fibrils and is systematically observed under conditions for accelerated fibrillation, indicating that the oligomers can act as on-pathway intermediates during amyloidogenesis [50]. It is interesting to note, that 5 PD patients and 2 controls exhibited the significant immune responses towards the oligomeric structures, which suggests that the latter may be populated in the sera of some individuals. Furthermore, in our pilot experiment we observed the immune reactivity towards amyloid fibrils in the pooled blood sera of 7 young controls of 20–25 years of age (Figure 3(C)), though at significantly lower level than in older controls and PD patients. As the presence of amyloid deposits in healthy individuals of this age group is highly unlikely, it cannot be ruled out that the amyloids can be formed transiently and the autoimmunity plays protective role as a natural clearance mechanism. The normal aging, however, may greatly increase the chances of protein misfolding and amyloid assembly, which puts a higher pressure on the clearance system and correlates with an enhanced level of autoimmune responses to α-synuclein amyloid fibrils even in the normal aging as well as during disease development (Figures 5, 3(C)).

As amyloid structures exhibit an inherent heterogeneity and mutual inter-conversion, we conducted our research on freshly prepared protein amyloid samples characterized by AFM, the thioflavin-T and Congo red biding, CD, as well as WST1 cytotoxicity assays (Figure 1, S1). Within our experimental set-up the amyloid oligomers were characterized by round-shape structure, weak binding of amyloid-specific dyes such as Congo red and thioflavin-T, lack of characteristic β-sheet spectrum in the far UV CD, though they interacted specifically with generic anti-oligomeric A11 antibodies and displayed pronounced cytotoxicity. In the previous study by using ELISA method El-Agnat *et al.* reported the presence of “soluble aggregates” or oligomers of α-synuclein in the plasma of PD patients [31]. As there are no further characteristics of these aggregates apart from their separation by size exclusion chromatography, it cannot be excluded that the oligomers subjected as antigens in our analysis (Figure 1B) and the “soluble aggregates” reported in [31] may belong to different types of pre-fibrillar or even fibrillar structures and possess different epitopes.

It is important to note that in the blood sera of PD patients the immune reactivity towards amyloid oligomers and fibrils of insulin also was not detected in the previous studies, though there were elevated autoimmune reactions to both insulin and S100B - endocrine and astrocytical biomarkers, respectively [9]. By contrast, in AD patients a significantly increased level of serum antibodies to both prefibrillar amyloids of Aβ and human lysozyme was found in the early stage of disease [8] and biphasic antibody levels to Aβ peptide oligomers - during the progression of dementia [7]. The autoimmune responses to Aβ oligomers reflected mild to moderate phases of AD dementia, while those to S100B protein closely matched moderate to severe dementia [7]. This suggests that the autoimmune reactivity and neurodegeneration in PD and AD may involve different underlying mechanisms.

**Figure 7:**
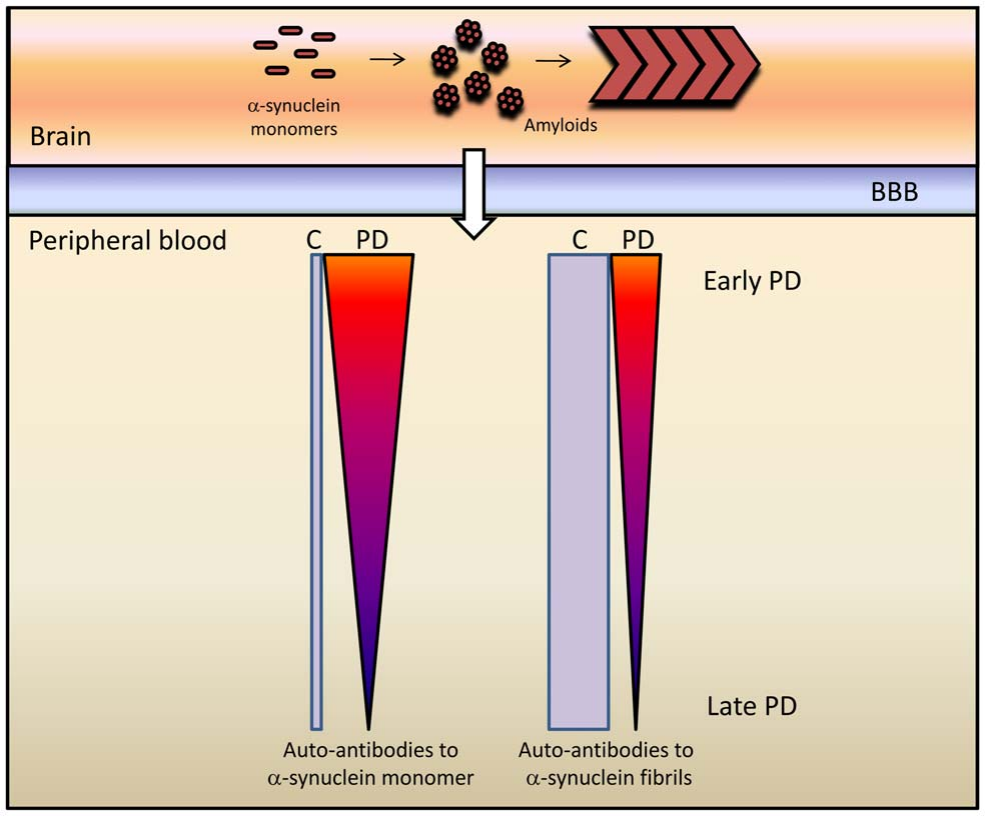
Schematic presentation of changes in the immune reactivity towards α-synuclein and its amyloids in the blood sera of PD patients and controls (C). The thickness of the bars and gradients is proportional to immune responses.

The trends of the autoimmune reactivity towards α-synuclein and its amyloids fibrils in the blood sera during progression of PD are summarized in Figure 7. Our findings indicate that the autoimmune response to α-synuclein can serve as a valid biomarker, reflecting the progressive brain neurodegeneration and impaired α-synuclein homeostasis occurring in PD. These findings, together with assessment of other biomarkers such as the autoimmune responses to insulin and S100B [9], may complement clinical investigations and be of importance as an aid for improved diagnostic accuracy of PD and as a tool to assess evolution of the disease as well as effects of interventions.

## Materials and Methods

### Ethics statement

All participants in this research gave informed and written consent. The study obtained ethics approval by the Umeå medical ethics committee of the Umeå University and Hospital.

### Human subjects

39 PD patients (24 males and 15 females) with a mean age of 63.3 years were recruited from the outpatient clinic of the Department of Neurology at Umeå University Hospital. The characteristics of the participants are presented in Table 1. Patients have been neurologically examined at the Department on several occasions and diagnosed as having clinically definite PD according to the UK Parkinson's Disease Society Brain Bank clinical diagnostic criteria [51]. Severity was assessed by the Hoehn and Yahr score [52] and the patients were divided into 2 groups according to the severity of disease: 27 patients were in a less advanced stage with 1 to 2 score (early PD) and 12 patients in a more advanced stage of 2.5–4 score according to Hoehn and Yahr scale (late PD). In 56% (22/39) of the patients the function of presynaptic dopamine system was investigated by FP-CIT SPECT imaging. All 22 patients showed reduced uptake of ligand in the putamen, as expected in PD and other forms of idiopathic parkinsonism. 2 patients were operated with deep brain stimulation (DBS) for treatment of their PD 5 and 8 years prior to the collection of blood for this study. 6 patients had a family history of movement disorders, mainly tremor. It was not possible to examine their relatives for a more exact diagnosis. 3 patients were treated for high blood pressure and another 3 patients had diabetes type 2. Patients with concomitant neurological or psychiatric diseases, cancer and other severe diseases were excluded. 23 healthy controls, biologically unrelated to the patients and of similar age and sex distribution as these, were selected from spouses of patients attending the outpatient clinic. The exclusion criteria for controls were identical to that of patients.

**Table 1:** Group characteristics of PD patients and controls.

	PD patients (n = 27)Hoehn/Yahr 1–2	PD patients (n = 12)Hoehn/Yahr 2.5–4	PD patients (n = 39)Total	Controls(n = 23)
Gender male/female	17/10	7/5	24/15	16/7
Age at PD onset	53.9±10.1	59.5±9.5	55.7±10.2	
Age at blood sample	60.6±10.9	69.5±7.0	63.3±10.6	57.4±13.8
Duration of PD	6.7±5.3	9.9±5.8	7.7±5.6	
UPDRS|– motor scale	22.2±12.4	36.4±13.7	25.9±14.0	

|– UPDRS – Unified Parkinson's Disease Rating Scale.

### α-synuclein production

The *Escherichia coli* BL21 (DE3) cells transformed with pRK173 plasmid harbouring the α-synuclein gene were used for the production of the recombinant protein [53]. The recombinant protein was purified as previously described [54] with some modifications outlined below. Plated cultures were used to inoculate Nutrient Broth medium (Oxoid Ltd, UK) containing ampicillin. Cultures were grown until late log-phase (A_600 nm_, 0.8) at 30°C and the protein expression was induced with 0.5 mM isopropyl-β-D-thiogalactopyranoside. The cells were cultured at 30°C overnight, harvested by centrifugation (3000 *g*, 20 min), washed, re-suspended in 50 mM Tris-HCl buffer, pH 7.5, containing 0.1 mM EDTA, 0.2 mM PMSF and disrupted by sonication. The cell homogenate was boiled for 10 min, the cell-free extract was loaded onto a HiPrep™ Q FF 16/10 Column (GE Healthcare) in 20 mM Tris-HCl, pH 7.5, and eluted by a linear 0–1 M NaCl gradient. Fractions containing α-synuclein were analyzed by a Coomassie stained SDS-PAGE and dialized against 20 mM Tris, pH 7.5. Collected fractions were loaded onto a HiTrap ANX FF (high sub) column and eluted by a linear 0–1 M NaCl gradient. Fractions containing α-synuclein were combined, dialized against 10 mM NH_4_HCO_3_ and lyophilized.

### Amyloid preparation

α-synuclein concentration was determined by optical absorbance measurements at 280 nm (ND-1000 spectrophotometer, Nano-drop, Sweden), using an extinction coefficient E_1 mg/ml_ = 0.354 [55]. In order to produce amyloid oligomers and fibrils of α-synuclein, protein was incubated at 0.21 mM and 0.71 mM concentrations, respectively, in 10 mM sodium phosphate buffer, pH 7.4 and 37°C, using continuous agitation at 300 rpm. Amyloid fibrils of Aβ (1–40) peptide were produced in 10 mM NaOH, pH 7.5 and room temperature after 3 days of incubation by using protocols described previously [56], while the fibrils of hen egg white lysozyme were formed in 20 mM glycine buffer at pH 2.2 and 57°C after 14 days of incubation [57], [58].

### Spectroscopic amyloid assays

Thioflavin T (ThT) binding assay was performed by using the modification of LeVine's method [59]. Thioflavin T fluorescence was measured by a Jasco FP-6500 spectrofluorometer (Jasco, Japan), using excitation at 440 nm and collecting emission between 450–550 nm, with excitation and emission slits set at 3 nm width. Congo red assay was performed as described in [57] by using a ND-1000 spectrophotometer for optical absorbance measurements. CD measurements were performed by using a Jasco J-810 spectropolarimeter (Jasco, Tokyo, Japan) equipped with a Jasco CDF-426L thermostat, using 0.1- and 0.5-cm path length cuvettes. At least three scans were averaged for each spectrum.

### Atomic force microscopy (AFM)

AFM measurements were performed on a PICO PLUS microscope (Agilent, USA) in a tapping mode as outlined previously [37], [60]. A scanner with a 100 µm scan size and acoustically driven cantilevers carrying etched silicon probes of the TESP model of 10 nm diameter (Veeco, Netherlands) were used. Typically we applied a resonance frequency in the 312–340 kHz range, scan rate of 1 Hz and a resolution of 512×512 pixels. Height, amplitude and phase data were collected simultaneously. Images were flattened and plane adjusted. The scanning of samples was performed in trace and retrace to avoid the scan artifacts. The scanner was calibrated by measuring atomic steps on highly orientated pyrolytic graphite in the *z*-axis and using a standard 1-µm calibration grid (Agilent, USA) in the *xy*-plane. Amyloid samples were deposited on the surface of freshly cleaved mica (GoodFellow, UK) for 30 min, washed three times with 100 µl of MilliQ water, and dried at room temperature. To determine the dimensions of amyloid species cross-section analysis in the height images was carried out using PICO PLUS software (Agilent, USA).

### Enzyme linked immunosorbent assay (ELISA)

The titers of serum antibodies to α-synuclein monomers and oligomers were determined by ELISA in 96-well polystyrol plates (Costar, USA) using 6 repetitions per sample. Microtiter wells were coated with the α-synuclein antigens at concentrations of 15.0 and 20.0 µg/ml of monomers and oligomers, respectively, in 50 mM phosphate buffer, pH 8.0 (4°C) and assayed by the protocol described in [8], [9]. The titer of each serum sample was derived from the reciprocal of the largest dilution at which the enzyme-substrate reaction gave an optical density value greater than that of the mean optical density of blanks (0.1 optical density). The values of titers at which we observed immunoreactivity to the antigens were represented in relative dilution ratio units.

### Electrophoresis and immunoblotting

Gel electrophoresis was performed under reducing conditions by using 15% SDS-PAGE gels to analyze the samples of freshly dissolved α-synuclein. Protein solutions were mixed with SDS loading buffer prior applying to SDS-PAGE. Larger molecular weight species such as α-synuclein amyloid oligomers and fibrils were separated by using native 8–25% gradient gels (Phast gels, GE Healthcare, Sweden). Coomassie brilliant blue R250 (Sigma, USA) was used for gel staining. Pre-stained molecular weight standards “SeeBlue” (Invitrogen, USA) were included in each experiment.

Immunoblotting was performed by using nitrocellulose membranes (0.45 µm, GE Healthcare, Sweden). Samples of monomeric α-synuclein and its amyloid species were transferred to membranes at 200 mA during 3 h and blocked with 5% milk in Tris buffered saline (TBS) containing 0.1% of Tween 20. Immune-reactivities between monoclonal antibodies towards α-synuclein (Abcam, UK), polyclonal antibodies towards amyloid oligomers and fibrils [5], [6] and respective antigens were used as positive controls in each experiment. Blood sera samples from PD patients and controls were applied to identify primary IgG interactions with α-synuclein antigens. Peroxidase-conjugated anti-human IgGs were used as secondary antibodies and their immune-reactivity was detected by using the enhanced chemiluminescence method (GE Healthcare, Sweden). Dot blot experiments were performed following the same procedure; we used 1 to 3 µg of freshly dissolved monomeric proteins and 3 to 4 µg of fibrillar materials per dot blot. The immune responses observed in the immunoblot analysis for individual PD patients and healthy controls were quantified densitometrically by using a Scion image software (NIH). Anti-oligomeric A11 [5] and anti-fibirllar [6] antibodies were a gift from Rakez Kayed.

### IgG Purification

Total IgG fraction from the blood sera was purified by using a Melon Gel Purification kit (Pierce Biotechnology, USA) according to the manufacturer's protocol. Purity of the IgG antibodies was assessed by 15% SDS PAGE.

### Biacore

The interaction between purified pools of IgGs from the blood sera and α-synuclein antigens were examined by a Biacore 3000 surface plasmon resonance instrument equipped with a CM4 sensor chip (GE Healthcare, Sweden). Monomers, oligomers and fibrils of α-synuclein were covalently immobilized in the flow cell with a target level of 10,000 RUs at 0.1 mg/ml concentration in 10 mM sodium acetate, pH 3.0, at room temperature. Total pooled IgGs were injected at 0.5 mg/ml concentration in filtered, degassed 0.01 M HEPES buffer, 0.15 M NaCl, 0.005% surfactant P20, pH 7.4 (GE Healthcare, Sweden) at a flow rate of 10 µl/min. The signal at the equilibrium level of the dissociation curve was taken as the measure of antigen-antibody interactions to exclude the non-specific interactions of all pooled IgGs with the Biacore chip [61].

### WST-1 cell viability assay

The neuroblastoma cell line SH-SY5Y [62] was used as in the previous studies Gharibyan *et al.* [58] and was originally purchased (Sigma, USA). This cell line were cultured in a Dulbecco's modified Eagle's medium supplemented with 10% (v/v) fetal bovine serum and antibiotics in a 5% CO_2_ humidified atmosphere at 37°C. Cells were plated at a density of 10^4^ cells/well in 96-well plates, incubated for 24 hours, and then the medium was changed before incubation with α-synuclein amyloid species. The α-synuclein samples were initially diluted in the culture medium and then added to the cells to achieve final concentration of 50 µM as described previously [58], [60]. Untreated cells as well as cells treated with the amyloid incubation buffer (10 mM sodium phosphate, pH 7.4) were used as controls.

In order to evaluate cell viability, 10 µl of water-soluble tetrazolium salt (WST-1) reagent was added per 100 µl of cell culture and samples were incubated at 37°C for 4 hours. Absorbance was measured with an ELISA plate reader (Tecan, Sweden) at 450 nm. Cell viability was expressed as a percentage of the absorbance in the wells containing cells treated with amyloid compared to the control wells. The experiments were performed in triplicate in each series and the results were presented as mean±SEM.

### ROC analysis

ROC analysis was used to quantify the potency of the ELISA and Western blot screening to discriminate between the autoimmune responses to α-synuclein in PD patients and controls. The ROC curves were plotted as the number of true-positives (sensitivity) *vs.* the number of false-positives (1-specificity) for all possible cut-off values. The areas under ROC curves (AUC) were calculated to evaluate the diagnostic values of the marker. If the test is without diagnostic power, the ROC curve will be linear with AUC of 0.5, (shown by dotted line in Figure 4). A perfect test would give an AUC of 1.0, indicating that it has zero false positives and zero false negatives. The higher AUC above 0.5, the better the test. For AUC>0.5, the choice of cut-off is a trade-off between the risk of missing true positives and of selecting false positives. Here we used approach based on the Youden index, J, calculated as maximum sensitivity + specificity −1. J corresponds to the maximum vertical distance between the ROC curve and the diagonal or chance line and it occurs at the cut-off point when equal weight is given to sensitivity and specificity [63], [64]. Statistical calculations were performed using the statistical package built into the GraphPad Prism 5 computer programme for the Macintosh (GraphPad Software Inc., San Diego, CA,USA).

### Statistical analysis

Statistical significance of measurements was assessed by using a non-parametric Mann Whitney *U* test and by a two-tailed Student *t*-test. The level of significance was set at *P*<0.05 (*** = *P*<0.0001, ** = *P*<0.007, * *P*<0.05).

## Supporting Information

Figure S1 Viability of SH-SY5Y cells treated with α-synuclein amyloids and measured by using WST-1 assay. Percentage of viable treated cells compared to untreated cells is shown in *y*-axis and the samples added to cells are indicated along *x*-axis. Cell viability was measured after 24 h (blue bars) and 48 h (red bars) of co-incubation with amyloids, respectively. ***P<0.0001.
(TIF)

Figure S2 SDS-PAGE of freshly dissolved α-synuclein. Reference molecular makers are shown in lane 1 and freshly dissolved α-synuclein with molecular mass of ca. 16 kDa – in lane 2.
(TIF)

## References

[1] Falk RH, Comenzo RL, Skinner M (1997). The systemic amyloidoses.. N Engl J Med.

[2] Kahn SE, Andrikopoulos S, Verchere CB (1999). Islet amyloid: a long-recognized but underappreciated pathological feature of type 2 diabetes.. Diabetes.

[3] Martin JB (1999). Molecular basis of the neurodegenerative disorders.. N Engl J Med.

[4] Selkoe DJ (1997). Alzheimer's disease: genotypes, phenotypes, and treatments.. Science.

[5] Kayed R, Head E, Thompson JL, McIntire TM, Milton SC (2003). Common structure of soluble amyloid oligomers implies common mechanism of pathogenesis.. Science.

[6] Kayed R, Head E, Sarsoza F, Saing T, Cotman CW (2007). Fibril specific, conformation dependent antibodies recognize a generic epitope common to amyloid fibrils and fibrillar oligomers that is absent in prefibrillar oligomers.. Mol Neurodegener.

[7] Gruden MA, Davidova TB, Malisauskas M, Sewell RD, Voskresenskaya NI (2007). Differential neuroimmune markers to the onset of Alzheimer's disease neurodegeneration and dementia: autoantibodies to Abeta((25–35)) oligomers, S100b and neurotransmitters.. J Neuroimmunol.

[8] Gruden MA, Davudova TB, Malisauskas M, Zamotin VV, Sewell RD (2004). Autoimmune responses to amyloid structures of Abeta(25–35) peptide and human lysozyme in the serum of patients with progressive Alzheimer's disease.. Dement Geriatr Cogn Disord.

[9] Wilhelm KR, Yanamandra K, Gruden MA, Zamotin V, Malisauskas M (2007). Immune reactivity towards insulin, its amyloid and protein S100B in blood sera of Parkinson's disease patients.. Eur J Neurol.

[10] O'Nuallain B, Hrncic R, Wall JS, Weiss DT, Solomon A (2006). Diagnostic and therapeutic potential of amyloid-reactive IgG antibodies contained in human sera.. J Immunol.

[11] Bard F, Cannon C, Barbour R, Burke RL, Games D (2000). Peripherally administered antibodies against amyloid beta-peptide enter the central nervous system and reduce pathology in a mouse model of Alzheimer disease.. Nat Med.

[12] DeMattos RB, Bales KR, Cummins DJ, Dodart JC, Paul SM (2001). Peripheral anti-A beta antibody alters CNS and plasma A beta clearance and decreases brain A beta burden in a mouse model of Alzheimer's disease.. Proc Natl Acad Sci U S A.

[13] Hock C, Konietzko U, Papassotiropoulos A, Wollmer A, Streffer J (2002). Generation of antibodies specific for beta-amyloid by vaccination of patients with Alzheimer disease.. Nat Med.

[14] Schenk D, Barbour R, Dunn W, Gordon G, Grajeda H (1999). Immunization with amyloid-beta attenuates Alzheimer-disease-like pathology in the PDAPP mouse.. Nature.

[15] Lambert MP, Viola KL, Chromy BA, Chang L, Morgan TE (2001). Vaccination with soluble Abeta oligomers generates toxicity-neutralizing antibodies.. J Neurochem.

[16] Check E (2002). Nerve inflammation halts trial for Alzheimer's drug.. Nature.

[17] de Rijk MC, Tzourio C, Breteler MM, Dartigues JF, Amaducci L (1997). Prevalence of parkinsonism and Parkinson's disease in Europe: the EUROPARKINSON Collaborative Study. European Community Concerted Action on the Epidemiology of Parkinson's disease.. J Neurol Neurosurg Psychiatry.

[18] Linder J, Stenlund H, Forsgren L (2010). Incidence of Parkinson's disease and parkinsonism in northern Sweden: a population-based study.. Mov Disord.

[19] Spillantini MG, Schmidt ML, Lee VM, Trojanowski JQ, Jakes R (1997). Alpha-synuclein in Lewy bodies.. Nature.

[20] Schapira AH (1999). Science, medicine, and the future: Parkinson's disease.. BMJ.

[21] Liu S, Ninan I, Antonova I, Battaglia F, Trinchese F (2004). alpha-Synuclein produces a long-lasting increase in neurotransmitter release.. EMBO J.

[22] Dawson TM, Dawson VL (2003). Molecular pathways of neurodegeneration in Parkinson's disease.. Science.

[23] Bennett MC (2005). The role of alpha-synuclein in neurodegenerative diseases.. Pharmacol Ther.

[24] Uversky VN (2003). A protein-chameleon: conformational plasticity of alpha-synuclein, a disordered protein involved in neurodegenerative disorders.. J Biomol Struct Dyn.

[25] Conway KA, Lee SJ, Rochet JC, Ding TT, Williamson RE (2000). Acceleration of oligomerization, not fibrillization, is a shared property of both alpha-synuclein mutations linked to early-onset Parkinson's disease: implications for pathogenesis and therapy.. Proc Natl Acad Sci U S A.

[26] Danzer KM, Haasen D, Karow AR, Moussaud S, Habeck M (2007). Different species of alpha-synuclein oligomers induce calcium influx and seeding.. J Neurosci.

[27] Lundvig D, Lindersson E, Jensen PH (2005). Pathogenic effects of alpha-synuclein aggregation.. Brain Res Mol Brain Res.

[28] Sharma N, Brandis KA, Herrera SK, Johnson BE, Vaidya T (2006). alpha-Synuclein budding yeast model: toxicity enhanced by impaired proteasome and oxidative stress.. J Mol Neurosci.

[29] Kuwahara T, Koyama A, Koyama S, Yoshina S, Ren CH (2008). A systematic RNAi screen reveals involvement of endocytic pathway in neuronal dysfunction in alpha-synuclein transgenic C. elegans.. Hum Mol Genet.

[30] Auluck PK, Chan HY, Trojanowski JQ, Lee VM, Bonini NM (2002). Chaperone suppression of alpha-synuclein toxicity in a Drosophila model for Parkinson's disease.. Science.

[31] El-Agnaf OM, Salem SA, Paleologou KE, Curran MD, Gibson MJ (2006). Detection of oligomeric forms of alpha-synuclein protein in human plasma as a potential biomarker for Parkinson's disease.. FASEB J.

[32] Tokuda T, Salem SA, Allsop D, Mizuno T, Nakagawa M (2006). Decreased alpha-synuclein in cerebrospinal fluid of aged individuals and subjects with Parkinson's disease.. Biochem Biophys Res Commun.

[33] Lee PH, Lee G, Park HJ, Bang OY, Joo IS (2006). The plasma alpha-synuclein levels in patients with Parkinson's disease and multiple system atrophy.. J Neural Transm.

[34] Mollenhauer B, Cullen V, Kahn I, Krastins B, Outeiro TF (2008). Direct quantification of CSF alpha-synuclein by ELISA and first cross-sectional study in patients with neurodegeneration.. Exp Neurol.

[35] El-Agnaf OM, Salem SA, Paleologou KE, Cooper LJ, Fullwood NJ (2003). Alpha-synuclein implicated in Parkinson's disease is present in extracellular biological fluids, including human plasma.. FASEB J.

[36] Li QX, Mok SS, Laughton KM, McLean CA, Cappai R (2007). Plasma alpha-synuclein is decreased in subjects with Parkinson's disease.. Exp Neurol.

[37] Malisauskas M, Zamotin V, Jass J, Noppe W, Dobson CM (2003). Amyloid protofilaments from the calcium-binding protein equine lysozyme: formation of ring and linear structures depends on pH and metal ion concentration.. J Mol Biol.

[38] Hong DP, Han S, Fink AL, Uversky VN (2010). Characterization of the Non-Fibrillar alpha-Synuclein Oligomers.. Protein Pept Lett.

[39] Polinsky RJ, McRae A, Baser SM, Dahlstrom A (1991). Antibody in the CSF of patients with multiple system atrophy reacts specifically with rat locus ceruleus.. J Neurol Sci.

[40] Papachroni KK, Ninkina N, Papapanagiotou A, Hadjigeorgiou GM, Xiromerisiou G (2007). Autoantibodies to alpha-synuclein in inherited Parkinson's disease.. J Neurochem.

[41] Orr CF, Rowe DB, Mizuno Y, Mori H, Halliday GM (2005). A possible role for humoral immunity in the pathogenesis of Parkinson's disease.. Brain.

[42] Hirsch EC, Hunot S (2009). Neuroinflammation in Parkinson's disease: a target for neuroprotection?. Lancet Neurol.

[43] Stolp HB, Dziegielewska KM (2009). Review: Role of developmental inflammation and blood-brain barrier dysfunction in neurodevelopmental and neurodegenerative diseases.. Neuropathol Appl Neurobiol.

[44] Lee HJ, Patel S, Lee SJ (2005). Intravesicular localization and exocytosis of alpha-synuclein and its aggregates.. J Neurosci.

[45] Hong Z, Shi M, Chung KA, Quinn JF, Peskind ER (2010). DJ-1 and alpha-synuclein in human cerebrospinal fluid as biomarkers of Parkinson's disease.. Brain.

[46] Masliah E, Rockenstein E, Adame A, Alford M, Crews L (2005). Effects of alpha-synuclein immunization in a mouse model of Parkinson's disease.. Neuron.

[47] Monsonego A, Maron R, Zota V, Selkoe DJ, Weiner HL (2001). Immune hyporesponsiveness to amyloid beta-peptide in amyloid precursor protein transgenic mice: implications for the pathogenesis and treatment of Alzheimer's disease.. Proc Natl Acad Sci U S A.

[48] O'Nuallain B, Wetzel R (2002). Conformational Abs recognizing a generic amyloid fibril epitope.. Proc Natl Acad Sci U S A.

[49] O'Nuallain B, Freir DB, Nicoll AJ, Risse E, Ferguson N (2010). Amyloid beta-protein dimers rapidly form stable synaptotoxic protofibrils.. J Neurosci.

[50] Hoyer W, Cherny D, Subramaniam V, Jovin TM (2004). Rapid self-assembly of alpha-synuclein observed by in situ atomic force microscopy.. J Mol Biol.

[51] Gibb WR, Lees AJ (1989). The significance of the Lewy body in the diagnosis of idiopathic Parkinson's disease.. Neuropathol Appl Neurobiol.

[52] Hoehn MM, Yahr MD (1967). Parkinsonism: onset, progression and mortality.. Neurology.

[53] Conway KA, Harper JD, Lansbury PT (1998). Accelerated in vitro fibril formation by a mutant alpha-synuclein linked to early-onset Parkinson disease.. Nat Med.

[54] Hoyer W, Antony T, Cherny D, Heim G, Jovin TM (2002). Dependence of alpha-synuclein aggregate morphology on solution conditions.. J Mol Biol.

[55] Narhi L, Wood SJ, Steavenson S, Jiang Y, Wu GM (1999). Both familial Parkinson's disease mutations accelerate alpha-synuclein aggregation.. J Biol Chem.

[56] Peralvarez-Marin A, Mateos L, Zhang C, Singh S, Cedazo-Minguez A (2009). Influence of residue 22 on the folding, aggregation profile, and toxicity of the Alzheimer's amyloid beta peptide.. Biophys J.

[57] Morozova-Roche LA, Zurdo J, Spencer A, Noppe W, Receveur V (2000). Amyloid fibril formation and seeding by wild-type human lysozyme and its disease-related mutational variants.. J Struct Biol.

[58] Gharibyan AL, Zamotin V, Yanamandra K, Moskaleva OS, Margulis BA (2007). Lysozyme amyloid oligomers and fibrils induce cellular death via different apoptotic/necrotic pathways.. J Mol Biol.

[59] LeVine H (1993). Thioflavine T interaction with synthetic Alzheimer's disease beta-amyloid peptides: detection of amyloid aggregation in solution.. Protein Sci.

[60] Malisauskas M, Ostman J, Darinskas A, Zamotin V, Liutkevicius E (2005). Does the cytotoxic effect of transient amyloid oligomers from common equine lysozyme in vitro imply innate amyloid toxicity?. J Biol Chem.

[61] Ritter G, Cohen LS, Williams C, Richards EC, Old LJ (2001). Serological analysis of human anti-human antibody responses in colon cancer patients treated with repeated doses of humanized monoclonal antibody A33.. Cancer Res.

[62] Ross RA, Biedler JL (1985). Presence and regulation of tyrosinase activity in human neuroblastoma cell variants in vitro.. Cancer Res.

[63] Perkins NJ, Schisterman EF (2006). The inconsistency of “optimal” cutpoints obtained using two criteria based on the receiver operating characteristic curve.. Am J Epidemiol.

[64] Thors L, Bergh A, Persson E, Hammarsten P, Stattin P (2010). Fatty acid amide hydrolase in prostate cancer: association with disease severity and outcome, CB1 receptor expression and regulation by IL-4.. PLoS One.

