# Supplementary figures and images for: α-Synuclein Reactive Antibodies as Diagnostic Biomarkers in Blood Sera of Parkinson's Disease Patients
Source: PLoS One. 2011 Apr 25;6(4):e18513. doi: 10.1371/journal.pone.0018513 (PMC3081826; doi:10.1371/journal.pone.0018513)

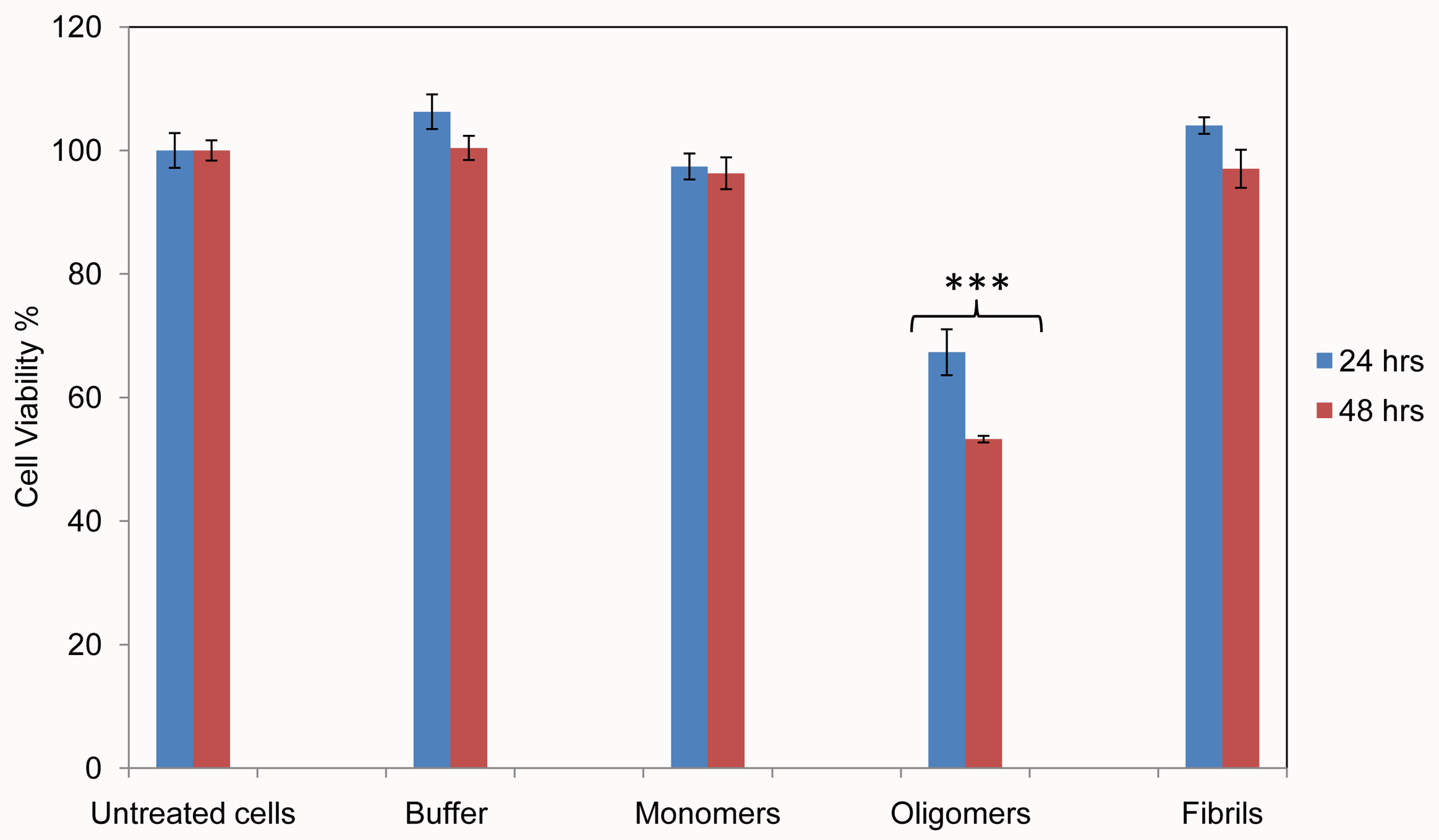

Supplement: Figure S1 — Viability of SH-SY5Y cells treated with α-synuclein amyloids and measured by using WST-1 assay. Percentage of viable treated cells compared to untreated cells is shown in y-axis and the samples added to cells are indicated along x-axis. Cell viability was measured after 24 h (blue bars) and 48 h (red bars) of co-incubation with amyloids, respectively. ***P<0.0001. (TIF) [file pone.0018513.s001.tif]

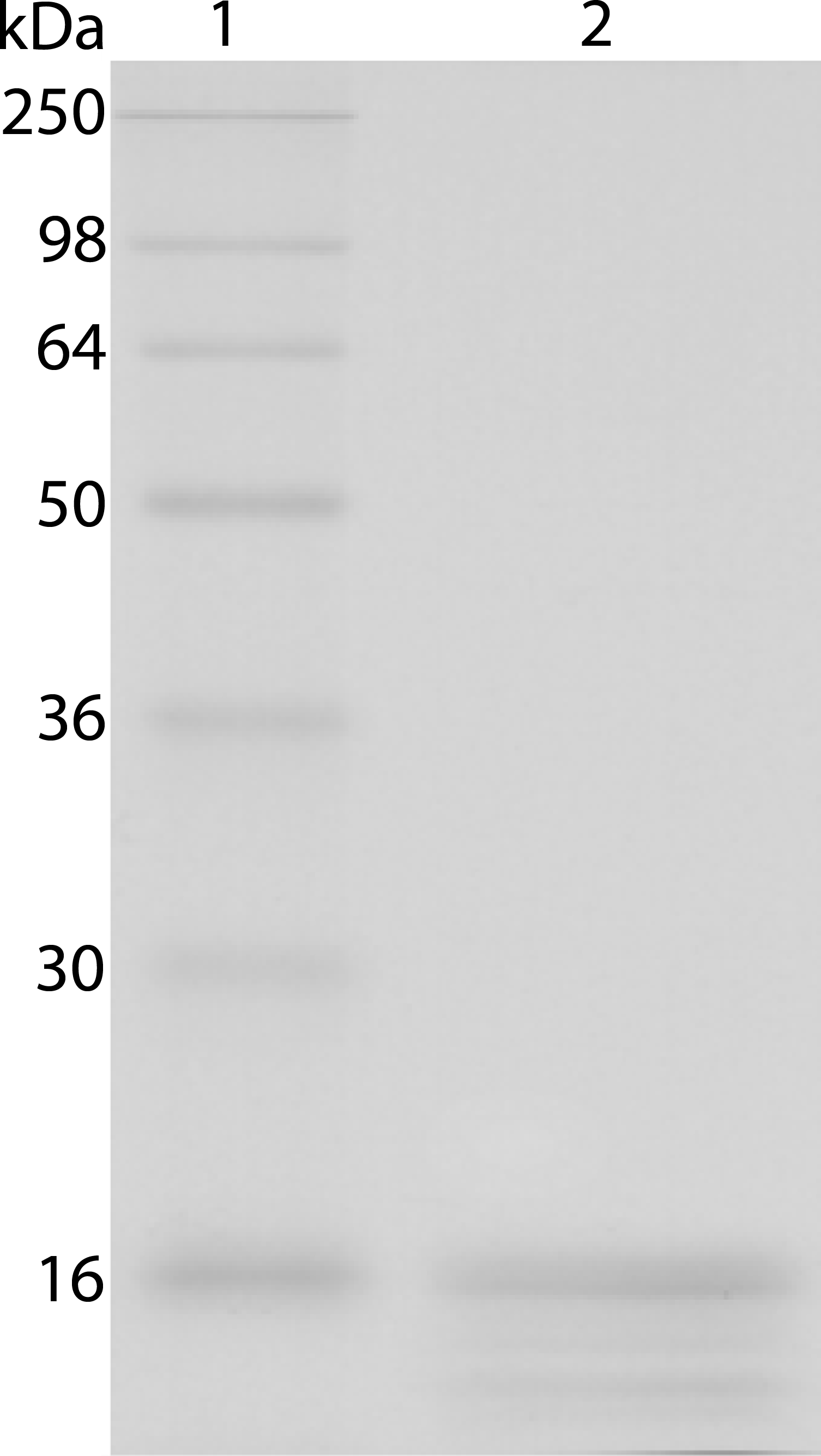

Supplement: Figure S2 — SDS-PAGE of freshly dissolved α-synuclein. Reference molecular makers are shown in lane 1 and freshly dissolved α-synuclein with molecular mass of ca. 16 kDa – in lane 2. (TIF) [file pone.0018513.s002.tif]
